# Mature dengue virus particles inactivated by a non-ionic detergent retain quaternary neutralizing epitopes and induce potent neutralizing antibodies

**DOI:** 10.3389/fimmu.2025.1626823

**Published:** 2025-09-10

**Authors:** Wen-Yang Tsai, Theodore C. Pierson, Jens Wrammert, Wanwisa Dejnirattisai, Amonrat Jumnainsong, Juthathip Mongkolsapaya, Gavin Screaton, James E. Crowe, Wei-Kung Wang

**Affiliations:** ^1^ Department of Tropical Medicine, Medical Microbiology and Pharmacology, John A. Burns School of Medicine, University of Hawaii at Manoa, Honolulu, HI, United States; ^2^ Vaccine Research Center, National Institute of Allergy and Infectious Diseases, The National Institutes of Health, Bethesda, MD, United States; ^3^ Emory Vaccine Center, Division of Infectious Disease, Department of Pediatrics, Emory University School of Medicine, Atlanta, GA, United States; ^4^ Nuffield Department of Medicine, Wellcome Trust Centre for Human Genetics, University of Oxford, Oxford, United Kingdom; ^5^ The Centre for Research and Development of Medical Diagnostic Laboratories, Faculty of Associated Medical Sciences, Khon Kaen University, Khon Kaen, Thailand; ^6^ Dengue Hemorrhagic Fever Research Unit, Office for Research and Development, Siriraj Hospital, Faculty of Medicine, Mahidol University, Bangkok, Thailand; ^7^ Division of Medical Sciences, John Radcliffe Hospital, University of Oxford, Oxford, United Kingdom; ^8^ Department of Pediatrics, Pathology, Microbiology and Immunology, The Vanderbilt Center for Antibody Therapeutics, Vanderbilt University Medical Center, Vanderbilt University, Nashville, TN, United States

**Keywords:** mature particles, neutralizing epitopes, Tween 20, dengue virus, vaccine

## Abstract

**Introduction:**

The four serotypes of dengue virus (DENV1-4) are the leading cause of arboviral diseases in humans. Currently, developing a safe and effective DENV vaccine remains a challenge. Previously we reported potently neutralizing human monoclonal antibodies (mAbs) preferentially recognize mature DENV particles, on which there is limited access to the fusion loop (FL) epitope and no premembrane (prM) protein. As FL and prM antibodies are weakly- or non-neutralizing and contribute to antibody-dependent enhancement, mature DENV particles represent an ideal DENV vaccine candidate. Several inactivated flavivirus vaccines, generated using formalin, ultraviolet or other inactivation methods, have progressed through preclinical and clinical studies. Little is known about how different inactivation methods affect viral epitopes and the quality of antibodies induced.

**Methods:**

We investigated epitopes on Tween 20-inactivated mature DENV1 particles by testing a panel of well-characterized human mAbs and membrane integrity by sucrose-gradient ultracentrifugation and protein K digestion. We examined the mechanisms of Tween 20 inactivation by measuring RNA copy numbers, virus binding to cells and acid exposure, and antibody responses induced by Tween 20-inactivated mature DENV1 particles in mice.

**Results:**

Tween 20 inactivation better preserved the epitopes recognized by potently neutralizing mAbs compared with other methods. Sucrose-gradient ultracentrifugation and protein K digestion revealed no disruption of membrane integrity by Tween 20. Mechanistically, Tween 20 treatment caused reduced virus binding to cells and RNA degradation, which was reverted by pre-treatment with RNAseOUT, suggesting the involvement of extracellular RNase, and prevented the envelope protein conformational changes induced by acid exposure. Moreover, Tween 20-inactivated mature DENV1 particles induced a neutralizing antibody response to all four DENV serotypes characterized by competition with several potently neutralizing mAbs and limited recognition of FL.

**Conclusion:**

Our results suggest that Tween 20-inactivated mature particles are a promising strategy for DENV vaccine development.

## 1 Introduction

During the past few decades, dengue has become the most important mosquito-borne viral disease in humans due to the co-circulation of four serotypes of dengue virus (DENV1−DENV4) in the tropical and subtropical areas along with the geographic expansion of mosquito vectors, *Aedes aegypti and Aedes albopictus* (1–3). It has been estimated that approximately 390 million infections, including 96 million apparent infections and 2 million severe cases, occur annually (1). Three tetravalent live-attenuated DENV vaccines have been tested in phase 3 clinical trials. Dengvaxia^®^ (CYD-TDV), developed by Sanofi Pasteur, was the first approved dengue vaccine with an overall efficacy of 59.2%, and was recommended for baseline DENV-immune individuals due to increased risk of severe disease among baseline DENV-naïve individuals (4–6). Dengvaxia was licensed in the US, EU, and some Asian and Latin American countries but will be discontinued in 2025 due to low global market demand (7). Qdenga^®^ (TAK-003), developed by Takeda, was the second approved dengue vaccine with an overall efficacy of 73.3%, albeit no efficacy against DENV3 and no data for DENV4 in baseline DENV-naive individuals (8–10). Qdenga has been licensed in the EU, UK, Brazil and other countries, but was withdrawn from the US license application (10). The Butantan-Dengue Vaccine had an overall efficacy of 79.6% in a phase 3 clinical trial, although efficacy data is not available for DENV3 or DENV4 (11), and this vaccine candidate has not yet been licensed. These findings together underscore the need of continued exploration of other platforms for DENV vaccines.

The RNA genome of DENV encodes a polyprotein, which is cleaved into three structural proteins, capsid (C), premembrane (prM), envelope (E), and seven non-structural proteins (12). The ectodomain of E protein, present on the surface of virion, contains three domains. Domain I (DI) is in the center, domain II (DII), which contains the fusion loop (FL) at its tip, is involved in dimerization and membrane fusion, and domain III (DIII), together with carbohydrates and lipids, is believed to be involved in receptor binding and stabilization of trimers during fusion (12–14). Several potently neutralizing human monoclonal antibodies (mAbs) have been reported, including those recognizing DIII residues and quaternary epitopes, such as the DI/DII hinge (DI/IIh) and E dimer epitope (EDE1 or EDE2) (15–21). Another type of human mAbs recognizes the highly conserved residues in the FL and/or bc loop of DII, known as FL antibodies (22, 23).

Cryo-EM studies revealed that populations of DENV virions consist of a mixture of mature, immature and partially immature particles (24–28). The maturation status of flavivirus particles has been shown to affect the epitope accessibility and thus the potency of neutralizing mAbs (29, 30). As inducing potently neutralizing antibodies against all four DENV serotypes, while reducing the risk of antibody-dependent enhancement (ADE), is an essential component of an ideal DENV vaccine, modulation of maturation status of DENV particles may have the potential to improve the development of safe and effective vaccines. A recent study reported that DENV circulating in humans had characteristics of mature DENV particles (mDENV); they were highly infectious and poorly neutralized by FL or bc loop mAbs compared with potently neutralizing mAbs including quaternary epitope and EDE2 mAbs (28). These findings underscore the importance of inducing potently neutralizing antibodies that can better neutralize DENV *in vivo* and provide protection. We reported previously that potently neutralizing human mAbs preferentially recognize mDENV particles, which lack prM protein and contain E protein dimers that limit the accessibility to the FL epitope (21, 26, 31, 32). Since FL and prM antibodies are weakly or non-neutralizing and have been shown to contribute to ADE *in vitro* and *in vivo* (33–38), mDENV particles may represent an ideal vaccine candidate to induce potently neutralizing antibodies and reduce the risk of ADE.

In addition to live-attenuated vaccines, other platforms that have been used for DENV vaccines in preclinical and clinical studies include the recombinant vaccine antigens such as protein subunit vaccine or virus-like particles (VLPs), viral vector-based vaccines, DNA or mRNA vaccines, and inactivated virus vaccines (39–47). For DENV and other flaviviruses, formalin-inactivated tick-borne encephalitis virus (TBEV) and Japanese encephalitis virus (JEV) vaccines have been demonstrated to exhibit their safety, immunogenicity and efficacy (48–51); formalin-inactivated Zika virus (ZIKV) and DENV vaccines are under development (40, 52, 53). One concern about formalin inactivation is that the cross-linking reaction can potentially damage or alter the antigenic structure of viral E protein, as supported by a report showing that a neutralizing epitope in the E protein DIII of JEV was altered by formalin (54). Another concern about formalin inactivation is that the process requires days to weeks to completely abolish virus infectivity. Other inactivation methods such as UV irradiation, H_2_O_2_ treatment, and psoralen-inactivation can efficiently inactivate viruses within minutes to hours (55–58). However, little is known about the extent to which different inactivation methods affect the neutralizing epitopes and the quality of antibodies induced by such inactivated antigens. Polysorbate 20, also known as Tween 20, is a nonionic detergent approved by the Food and Drug Administration for various applications in the food, biotech and pharmaceutical industries (59). For most immunoassays, Tween 20 can be added to washing buffers to reduce background signals and has been shown to inactivate infectious agents including viruses in human serum/plasma samples (60–62); however, it has never been exploited for vaccine development.

The objective of this study was to explore a new DENV vaccine strategy by inactivating mDENV particles with Tween 20. We investigated the epitope preservation of Tween 20 inactivated mDENV particles, mechanisms of action and immunogenicity in mice.

## 2 Materials and methods

### 2.1 Cells, mature virus particles

Vero cells stably expressing furin protease (Vero-furin) were used for production of mature virus particles (63, 64). The cells were maintained in DMEM (Gibco) supplied with 10% FBS, 2 mM HEPES, 1% penicillin-streptomycin and 50 µg/mL blasticidin (Invitrogen). After infection, the medium was replaced with DMEM supplied with 2 mM HEPES, 1% penicillin-streptomycin and 25 µg/mL blasticidin. The medium was collected every three days for three times. DENV1 Hawaii strain, DENV2 New Guinea C (NGC) strain, and ZIKV PRVABC-59 strain were used to generate mature particles. The relative prM content of mDENV particles generated, as determined by a previously reported virion ELISA (31), was very low compared with that of mixed and immature DENV particles (**Supplementary Figure 1**).

### 2.2 Human mAbs

The category, binding specificity, epitope and neutralizing potency of human mAbs used in this study were summarized in **Table 1** (17–22, 31, 65, 66).

**Table 1:** The category, binding specificity, epitope and neutralizing potency of human monoclonal antibodies in this study.

mAb	Category	Binding specificity*^a^*	Domain*^b^*	Epitopes*^b^*	NT_50_ *^c^* to D1 (D2, ZIKV)	Reference
82.11	FL	GR	I/II	W101	0.043	(17)
DV291.3	FL	GR	I/II	W101, F108	0.55	(17, 31)
DV143.6	FL	GR	I/II	L107, D290	0.50	(17, 31)
DVG1.3	FL	GR	I/II	L107	0.52	(22)
DVG12.2	FL	GR	I/II	L107, F108, T76	>2	(22)
DVC23.13	FL	GR	I/II	W101, F108	1.0	(22)
DV470.12	III	TS D1	III	G383, E384, K385, 79E	0.01	(17, 31)
DV87.1	III	CR D1-3	III	K307, E311, L389, W391	0.007	(17, 31)
DVG17.12	III	TS D1	III	G383, E384, K385	0.005	(22)
1F4	I/II hinge*^d^*	TS D1	I/II	K47, N52, K136, E157, T160, T161, T163, G274	0.11	(18)
747C4	EDE2*^d^*	CR	I/II	E49, Q77, W101, N153, T155, I161, P169, T200, W391, F392	0.030	(21)
747D8	EDE2*^d^*	CR	I/II	E49, Q77, W101, N134, N153, T155, I161, A162, P169, T200, K202, E203, L308, K310, Q323, W391, F392	0.021	(21)
752-2C8	EDE1*^d^*	CR	I/II	E49, Q77, W101, I161, A162, T200, Q323, W391, F392	0.131	(21)
752-2B2	EDE1*^d^*	CR	I/II	E49, Q77, W101, I161, A162, P169, T200, Q323, W391, F392	0.061	(21)
33.3A06	EDE1*^d^*	CR	I/II	W101, W391		(65, unpublished)
DVC3.7	III	TS D2	III	V382	<0.07 (D2)	(20)
DVC10.16	III	TS D2	III	E311	<0.08 (D2)	(20)
DVC25.5	III	TS D2	III	NA	0.3 (D2)	(20)
2D22	III q*^d^*	TS D2	III	R323	0.08 (D2)	(19)
ZIKV116	III [B] *^e^*	ZIKV,D1,D4	III	T309, E393, K394	0.016 (ZIKV)	(66)
ZIKV19	II [C]*^e^*	NT	II	W217, F218, D220, P222	>0.42 (ZIKV)	(66)
ZIKV195	(II) [D]*^e^*	NT	(II)	(D67, Q89, K118)	0.347 (ZIKV)	(66)
ZIKV117	II q [D]*^e^*	TS ZIKV	II	D67, Q89, K118	0.005 (ZIKV)	(66)
ZIKV88	(FL) [A]*^e^*	NT	I/II	(W101, F108)	>0.42 (ZIKV)	(66)

*^a^* GR: group-reactive to flaviviruses, CR: complex-reactive to DENV serocomplex, TS, type-specific; NT, not tested.

*^b^* Domain and epitopes were based on the location of the epitope residues determined by binding or escape mutants, or binding to different domains (DI/II or DIII) reported previously. NA, not applicable.

*^c^* NT_50_ values (μg/mL) to DENV1 (D1), DENV2 (D2) or ZIKV.

*^d^* I/II hinge, III q, EDE1 and EDE2, and II q mAbs are quaternary epitope mAbs.

*^e^* For ZIKV mAbs, competition groups [A, B, C, D] were determined (66). Data in parentheses were referred from competition group.

### 2.3 Virus inactivation

For formalin inactivation, 0.05% formaldehyde was used for crude virus culture supernatant at 37 °C for 24 h (67) and 0.02% formaldehyde purified virus at 22 °C for 10 days (68, 69). After inactivation, the remaining formalin was neutralized with 0.05% sodium bisulfite at room temperature for 15 min. For UV inactivation, the virus was exposed to 254 nm UV light (using a Stratagene Crosslinker) for 5 min and cooled down on ice for 1 min, followed by another exposure and cooling down. For Tween 20 inactivation, the crude virus supernatant was treated with 1% Tween 20 (Promega) at 37 °C for 1 h and shaken every 15 min. Excessive Tween 20 was removed in downstream purification steps.

### 2.4 Focus assay

Vero cells were seeding on flat-bottom 96-well plates at 3.5 x 10^4^ cells per well one day prior to infection. Fifty µL of 10-fold serial diluted untreated or post-inactivation virus supernatants were inoculated to each well, followed by 1.5 h incubation with gently shaking every 15 min (22). After adding 150 µL of 1% methylcellulose prepared in DMEM (Gibco) supplied with 2% FBS, 2 mM HEPES and 1% penicillin-streptomycin, the plates were incubated at 37 °C, 5% CO_2_ incubator for 48–70 h. After washing with PBS and fixation with ice-cold 70% acetone and 30% methanol at -20 °C for 20 min, the plates were air-dried, blocked with 5% non-fat milk in PBS for 30 min, and stained with murine anti-E mAb 4G2 or anti-DENV prM mAb 2H2, followed by HRP-conjugated secondary antibody (22). After adding KPL TrueBlue™ peroxidase substrates (SeraCare), virus foci were read by CTL ImmunoSpot Analyzer.

### 2.5 Virus purification

Crude virus culture supernatant was pre-cleared by low-speed centrifugation at 1,200 × g for 20 min and passed through a 0.22 µm syringe filter. Virus was concentrated by 20% sucrose cushion at 110,000 × g for 5 h at 4 °C. After resuspension in PBS, concentrated virus was diluted in starting buffer (20 mM Tris, 150 mM NaCl, pH 7.5) and further purified via HiTrap Capto Core 400 column (GE Life Sciences) in fast protein liquid chromatography (FPLC) using an ÄKTA pure™ chromatography system (Cytiva). Flowthrough fractions containing purified viruses were pooled and further concentrated with an Amicon Ultra centrifugal filter 100 kDa cut-off with PBS. Purified virus was further passed through 0.22 µm syringe filter and stored at 4 °C. The concentration of E protein in purified particles was determined by SDS 12% polyacrylamide gel electrophoresis (PAGE) including serial dilutions of BSA (from 2 µg to 0.0625 µg) as standards, followed by Coomassie blue staining. Protein intensity in stained gel was quantified in Odyssey scanner (Li-Cor).

### 2.6 Virion-capture ELISA

Virion-capture ELISA was performed as described previously with minor modifications (31). Briefly, mixed mouse mAbs 4G2 and FL0251 (700 ng/well) were coated on 96-well ELISA plates overnight. Crude virus culture supernatants with or without inactivation were 1:4 diluted in StartingBlock blocking buffer (Thermo Fisher Scientific) and added into each well. The plates were incubated at 37 °C for 2 h. After wash with ELISA washing buffer (0.5% Tween 20 in PBS), the plates were added with human mAb at 0.5−2 µg/mL at 37 °C for 2 h, followed by secondary (anti-human) antibody conjugated with HRP (Jackson Immunoresearch) at 37 °C for 1 h. After final wash, the plates were added with TMB substrate, followed by stop solution; the optical density at 450 nm was read with a reference wavelength of 650 nm.

### 2.7 Centrifugation and proteinase K digestion-protection assay

Virus culture supernatants were pretreated with or without 1% Tween 20 or 1% Triton X-100 at 37 °C for 1 h. Two mL supernatants were directly pelleted down in centrifuge at 22,500 × g for 2 h at 4 °C. Pellets were resuspended in PBS for Western blot analysis. For proteinase K digestion-protection assay (70), untreated or 1% Tween 20 inactivated virus pellets were further treated with or without 2% Triton X-100 for 10 min, followed by digestion with proteinase K (50 µg/mL) for 15 min on ice. The reaction was stopped by adding protease inhibitor cocktail (Roche), followed by adding 2× Laemmli sample buffer (Bio-Rad) for Western blot analysis.

### 2.8 Sucrose gradient sedimentation analysis

Virus culture supernatants pretreated with 1% Tween 20 or not were concentrated by 20% sucrose cushion ultracentrifugation at 110,000 × g for 5 h at 4 °C. The pellets were resuspended in PBS and loaded onto 15%−60% sucrose gradient, followed by ultracentrifugation at 38,000 × g for 18 h at 4 °C (71). After ultracentrifugation, each fraction was collected from top to bottom and analyzed by SDS 12% PAGE and Western blot analysis (31).

### 2.9 Quantitative real-time RT-PCR

This method was described previously (71). Briefly, extracted viral RNA and serial dilutions of a control plasmid consisting of DENV1 3′-nontranslated region with known copy numbers (as a standard) were quantified by a real-time RT-PCR assay using primers targeting the DENV1 3′-nontranslated region (5′-ACACCAGGGGAAGCTGTACCCTGG-3′ and 5′-CATTCCATTTTCTGGCGTTCT-3′) (72). The reaction was performed by using iSript™ one-step RT-PCR kit with SYBR Green (Bio-Rad) in Applied Biosystems™ 7500 Real-Time PCR Systems. The amount of viral RNA was expressed as genome equivalent (GE) copy number.

### 2.10 Cell-based virion binding ELISA

Vero cells were seeded onto 96-well plates at 3.5 x 10^4^ cells per well overnight. One µg E-protein-equivalent of purified DENV particles, DMEM medium with 10% FBS or BSA (20 µg/mL) were added to cells and incubated on ice for 2 h. After washing with PBS twice, the cells were fixed with ice-cold 4% paraformaldehyde on ice for 20 min. After PBS wash, the plates were blocked with ELISA blocking buffer and incubated with anti-E DIII human mAb 470.12 (0.5 µg/mL) at 37 °C for 2 h, followed by secondary antibody. The addition of TMB substrate and stop solution and the reading of OD was performed as regular ELISA described above.

### 2.11 Mouse immunization

Groups of 6–10 week-old BALB/c mice (n=9, 5 females, 4 males) were intraperitoneally injected with three doses of 1 µg E-protein-equivalent purified Tween 20-inactivated mDENV1 with 0.1% Alhydrogel (Invitrogen) at 0, 4 and 8 weeks. Blood was drawn from the submandibular vein at -1, 3, 7 and 11 weeks. Mice were sacrificed at 12 weeks. Blood was collected after euthanasia by cardiac puncture for the analysis of antibody response.

### 2.12 Virion ELISA and endpoint titers

Concentrated mDENV1 virion (Hawaii strain) were coated onto 96-well ELISA plates at 4 °C overnight, followed by blocking and incubation with serial diluted mouse sera (starting at a 1:400 dilution) and secondary antibodies (31). After a final wash and incubation with TMB substrate and stop solution, the OD at 450 nm was read with a reference wavelength of 650 nm. The cutoff was determined by the mean OD value obtained from pooled pre-immune mouse sera at a 1:100 dilution plus three standard deviations. The endpoint titer was determined as the reciprocal of the highest serum dilution reaching cutoff value calculated by using four parameters nonlinear regression analysis in Prism version 6 (GraphPad).

### 2.13 Microneutralization test

Microneutralization test was performed as described previously (21, 31). Briefly, flat-bottom 96-well plates were seeded with Vero cells (3 x 10^4^ cells per well) 24 h prior to infection. Fourfold serial dilutions of sera were mixed with 50–100 focus-forming units (FFU) of DENV1 (Hawaii strain), DENV2 (NGC strain), DENV3 (CH53489), or DENV4 (H241 strain) at 37 °C for 1 h. The mixtures were added to each well, followed by incubation for 48 to 70 h. After washing with PBS, the plates were fixed with ice-cold 30% methanol and 70% acetone, air-dried and blocked with 5% milk in PBS. After adding murine mAb 4G2 and a secondary antibody mixture (IRDye 800CW-conjugated goat anti-mouse IgG at 1:10,000 and the DRAQ5 fluorescent probe at 1:10,000) and washing with PBS, the plates were air-dried overnight. The intensity (800 nm/700 nm fluorescence) was detected by the LiCor Odyssey classic imaging system (LiCor Biosciences) and analyzed by Image Studio software to determine the intensity of each well. % of infection at different serum concentrations was calculated by the value of 800/700 ratio interpolated to the linear curve generated from virus-only controls with six dilutions (100%, 75%, 50%, 25%, 10% and 0%). % neutralization was calculated as 100% - % of infection. NT_50_ titer was the serum titer for 50% of neutralization determined by using four-parameter nonlinear regression analysis in Prism version 6 (GraphPad).

### 2.14 Blockade of binding ELISA

The method was performed as described previously (73). Fourfold serial dilutions of pooled mouse sera (starting at 1:400) were incubated with DENV1-capture ELISA plate. After washing, 0.25 µg/mL of human mAb was added followed by an anti-human secondary antibody conjugated with HRP. The ELISA plate was read as described above. The percentage of human mAb binding was calculated as OD of mAb with immune mouse sera/OD of mAb with pre-immune mouse sera. The reciprocal of a serum dilution that inhibits 50% binding of a human mAb was determined as IC_50_ titer.

### 2.15 Proportion of FL antibody

The proportion of FL antibody was determined by a capture ELISA using DENV1 wild-type (WT) and FL-mutant (W101A+F108A) VLPs as described previously (74). Briefly, 96-well plates were coated with rabbit anti-serum against DENV1 at 4 °C overnight, followed by blocking with 1% BSA in 1X PBS for 1 hour. VLPs (at ~0.01 μg/mL) were added, followed by two-fold serial dilutions of mouse immune sera, anti-mouse IgG conjugated to HRP, TMB substrate and stop solution (74). The absorbance was read as described above. The endpoint titers were the reciprocal of the highest titers that yielded a signal greater than 3 standard deviations of the mean signal from pre-immune sera. The proportion of FL antibody was determined by the formula: [1 - endpoint titer to mutant VLPs/endpoint titer to WT VLPs] X 100% (74).

### 2.16 Statistical analysis

The two-tailed Fisher’s exact test was used to compare qualitative variables between two groups. The two-tailed Mann-Whitney test and Wilcoxon rank signed test were used to compare quantitative variables between two groups and within a group, respectively (Prim version 6.0). The two-tailed Spearman correlation test was used to determine the relationship between NT_50_ and ELISA titers (Prism version 6.0).

## 3 Results

### 3.1 Tween 20 effectively inactivates DENV and ZIKV

**Figure 1:**
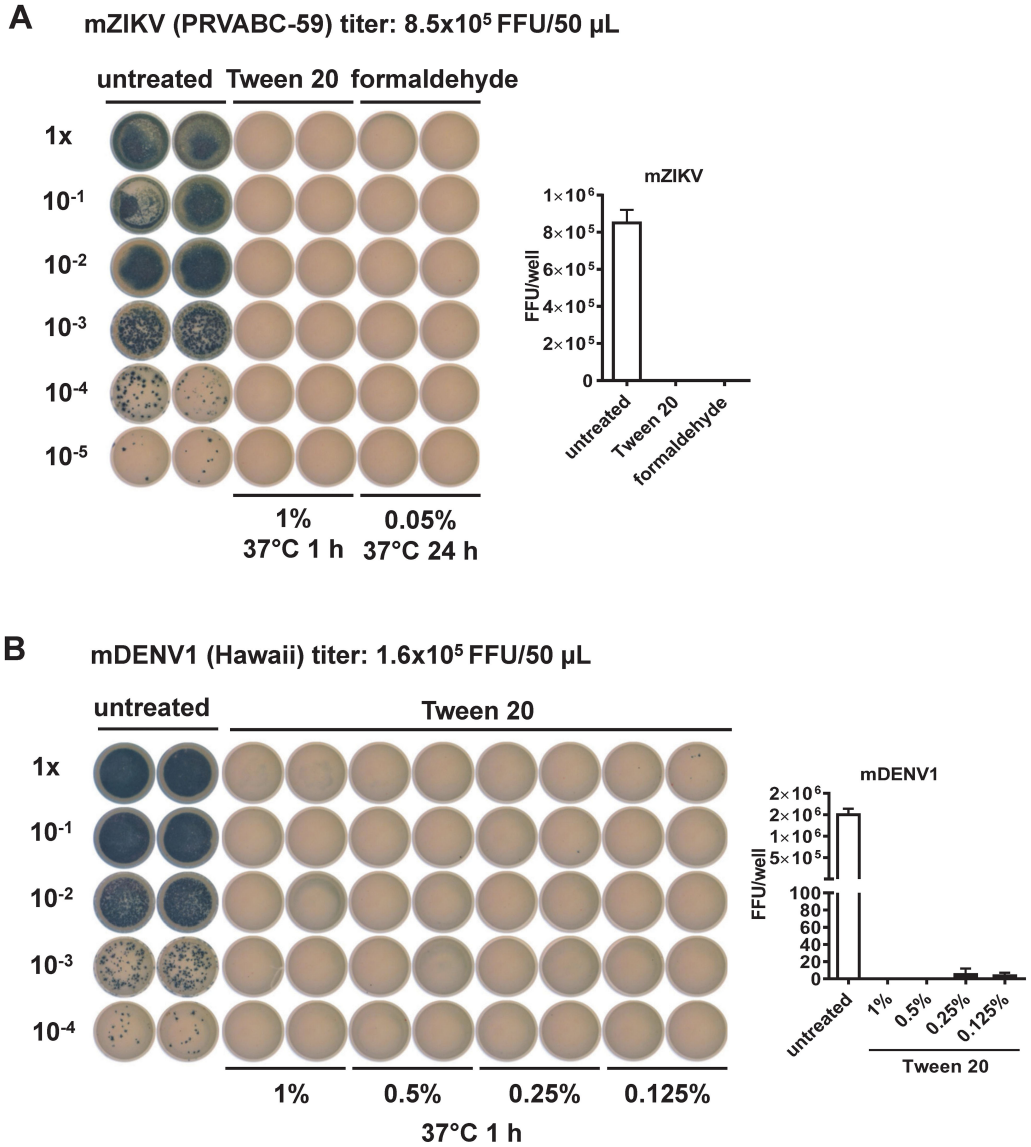
Evaluation of virus inactivation methods by the focus assay (22). **(A)** mZIKV (8.5x10^5^ FFU, PRVABC-59 strain) was inactivated by Tween 20 or formaldehyde compared to untreated control. Inactivated mZIKV particles were 10-fold serially diluted and inoculated into Vero cells in duplicates for 48 h, followed by fixation and staining with mouse anti-E mouse mAb (4G2), secondary antibody and TrueBlue as described in the Methods. FFU per well were shown at the right. **(B)** mDENV1 (1.6x10^5^ FFU, Hawaii strain) was inactivated by different concentrations of Tween 20, followed by the focus assay as above. FFU per well were shown at the right.

To evaluate the effectiveness of Tween 20 inactivation, we first treated mature preparations of ZIKV (mZIKV) virions with 1% Tween 20 at 37 °C for 1 h. Formaldehyde inactivation of mZIKV was performed in parallel as reported previously (0.05% at 37 °C for 24 h) (67–69). After inactivation, the focus assay did not detect any FFU at 48 h in either method, suggesting complete abolishment of virus infectivity (**Figure 1A**). To determine the lowest concentration of Tween 20 capable of inactivating infection, mDENV1 virions were treated with different concentrations; foci were not detectable following inactivation with Tween 20 at concentrations ≥0.5%, suggesting 0.5% is the lowest effective concentration (**Figure 1B**). A similar result was observed in mZIKV particles treated with differing concentrations of Tween 20 (Supplementary **Figure 2**). Furthermore, incubation with Tween 20 at 37 °C was more efficient at inactivation than what was observed at 22 °C (Supplementary **Figure 2**). To ensure complete inactivation for our vaccine candidate, we chose to proceed with 1% Tween 20 and 1 h incubation at 37 °C.

**Figure 2:**
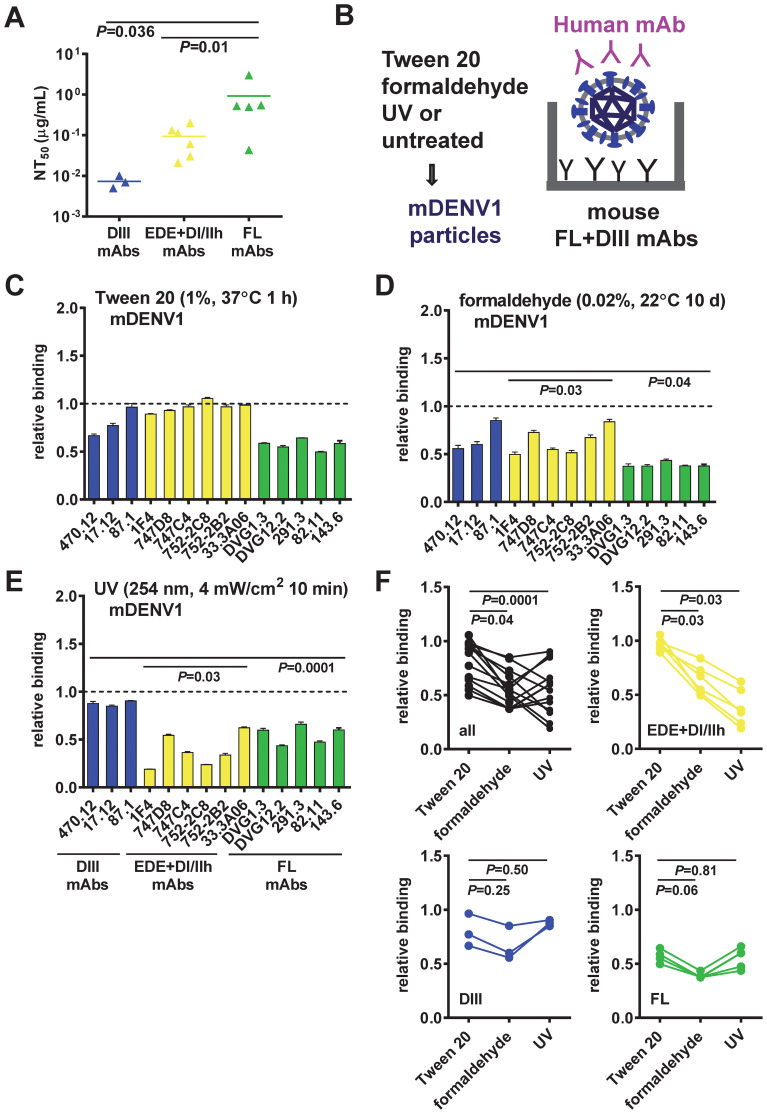
Epitope preservation of inactivated mDENV1 particles. **(A)** Neutralizing potency of three groups of human mAbs tested: DIII, quaternary epitope (EDE, DI/II hinge), and FL mAbs. The two-tailed Mann-Whitney test was used to compare two groups. **(B−E)** Virion-capture ELISA was used to assess the epitopes on mDENV1 particles inactivated by Tween 20 **(C)**, formaldehyde **(D)**, or UV **(E)**, using a panel of 14 human mAbs including three groups: DIII (blue), EDE and DI/II hinge quaternary epitope (yellow), and FL (green) mAbs. **(F)** The two-tailed Wilcoxon signed rank test was used to compare the relative binding of all and each group of mAbs to Tween 20-inactivated mDENV1 particles and to formaldehyde- or UV-inactivated particles. Significant *P* values were also shown in **(D, E)**. The relative binding was the OD of a mAb bound to inactivated mDENV1 particles relative to that of binding to untreated particles (relative binding=1). Data were means and standard deviations of triplicates from one representative experiment of two.

### 3.2 Tween 20 inactivation preserves neutralizing epitopes on mDENV and mZIKV particles

To evaluate epitope preservation on mDENV particles after inactivation by different methods, we used virion-capture ELISA to assess the recognition of inactivated mDENV1 particles by a panel of 14 well-characterized human mAbs that bind diverse epitopes, including three DIII-specific mAbs, six mAbs that bind quaternary epitope (three EDE1, two EDE2, and one DI/DII hinge mAbs), and five FL-reactive mAbs (17–22, 31, 65, 66) (**Table 1**). DIII mAbs were the most potently neutralizing, followed by the quaternary epitope mAbs and the FL mAbs (**Figure 2A**). Compared with the recognition of untreated virions, Tween 20-inactivated mDENV1 particles maintained a relative binding of 67-96% to DIII mAbs, 89-100% to quaternary epitope mAbs, and 50-64% to FL mAbs (**Figures 2B, C**). In contrast, formaldehyde-inactivated/UV-inactivated mDENV1 particles had a relative binding of 56-85%/85-90% to DIII mAbs, 50-84%/16-62% to quaternary epitope mAbs and 37-43%/44-62% to FL mAbs (**Figures 2D, E**). These findings suggest that Tween 20 inactivation better preserved the binding of mDENV1 particles to all 14 mAbs tested compared with formaldehyde or UV inactivation (relative binding to all mAbs: *P* = 0.04 or *P* = 0.0001, respectively, the two-tailed Wilcoxon signed rank test, **Figure 2F**). Subgroup analysis revealed that after Tween 20 inactivation the relative binding to quaternary epitope mAbs was higher than that after formaldehyde or UV inactivation (*P* = 0.03, both comparisons, the two-tailed Wilcoxon signed rank test, **Figure 2F**). To further confirm the epitope preservation after Tween 20 inactivation of other flaviviruses, we examined Tween 20-inactivated mDENV2 and mZIKV particles using additional mAbs. Tween 20 inactivation maintained the binding of mZIKV particles to all 8 mAbs tested including one DIII, two FL, two EDE1, two DII mAbs and one DII quaternary mAb (ZIKV-117) (**Table 1**, **Supplementary Figure 3A**) (66, 75). Similarly, Tween 20-inactivated mDENV2 particles did not disrupt the binding to all 13 mAbs tested (**Supplementary Figure 3B**); they actually increased the binding of several mAbs. This could be due to the irreversible formation of bumpy particles at 37 °C, as reported in previous cryo-EM studies of mDENV2 particles (76, 77), during the Tween 20 inactivation step; bumpy particles likely have more epitopes exposed and increased binding by different mAbs compared with untreated particles. Taken together, these results indicated that Tween 20 inactivation preserve neutralizing epitopes on mDENV1, mDENV2 and mZIKV particles.

### 3.3 Purification of Tween 20-inactivated mDENV particles preserves the neutralizing epitopes

**Figure 3:**
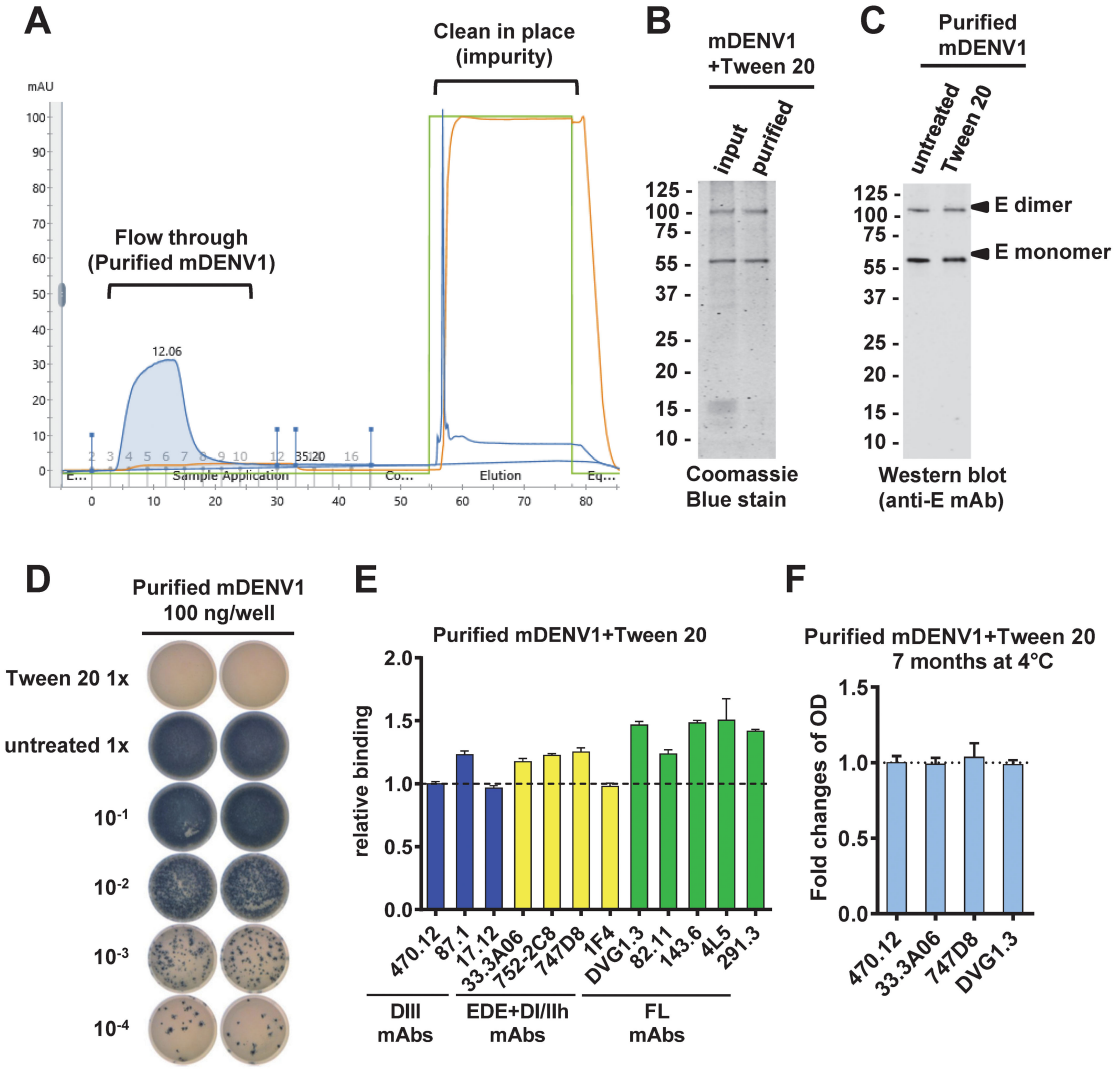
Purification of untreated and Tween 20-inactivated mDENV1 particles. **(A)** mDENV1 particles were concentrated by 20% sucrose cushion ultracentrifugation and purified by HiTrap Capto Core 400 column in AKTA pure system. **(B)** Input and purified (flow-through) particles were examined by SDS 12% PAGE and Coomassie blue staining (31). **(C)** Purified untreated and Tween 20-inactivated mDENV1 were detected by Western blot analysis using anti-E mouse mAb (4G2). **(D)** Focus assay was performed for purified untreated and Tween 20-inactivated mDENV1 (22). 1x represents 100 ng E-protein-equivalent of mDENV1. **(E)** Purified Tween 20-inactivated mDENV1 particles were examined by virion-capture ELISA using a panel of human mAbs including DIII (blue), EDE and DI/II hinge quaternary epitope (yellow) and FL (green) mAbs. The relative binding was the OD of a mAb bound to inactivated mDENV1 particles relative to that to untreated particles. **(F)** The stability of purified Tween 20-inactivated mDENV1 was examined by virion-capture ELISA using four representative human mAbs before and after storage at 4°C for 7 months. Data were means and standard deviations of quadruplicates from one experiment and presented as fold changes of OD over 7 months.

To purify mDENV particles after Tween 20 inactivation, we performed 20% sucrose cushion ultracentrifugation, followed by FPLC using an ÄKTA system (**Figure 3A**). Coomassie blue staining of a protein gel electrophoresis revealed higher purity of the flow-through mDENV1 particles compared with input particles (**Figure 3B**). Western blot analysis revealed comparable E protein bands in untreated and Tween 20-inactivated mDENV1 particles (**Figure 3C**). After purification, Tween 20-inactivated mDENV1 particles were not infectious, whereas untreated mDENV1 particles remained infectious (**Figure 3D**). Virion capture ELISA probing with a panel of human mAbs revealed that purified Tween 20-inactivated mDENV1 particles maintained the binding to all 12 human mAbs tested comparable to the findings with purified untreated mDENV1 particles, suggesting the purification process did not affect the epitope recognition of mDENV1 particles (**Figure 3E**). After storage at 4 °C for 7 months, the purified Tween 20-inactivated mDENV1 particles retained the capacity to bind all 4 of the representative human mAbs tested (470.12, 33.3A06, 747D8 and DVG1.3), suggesting high stability of the particles when stored at 4 °C for 7 months (**Figure 3F**).

### 3.4 Tween 20 inactivation does not affect the membrane integrity of mDENV particles

We next focused on mDENV1 particles in the following mode of action and immunogenicity studies, except for the sucrose cushion ultracentrifugation and proteinase K-protection experiments, which involved the detection of C protein by an available anti-DENV2 C mAb, we examined mDENV2 particles.

**Figure 4:**
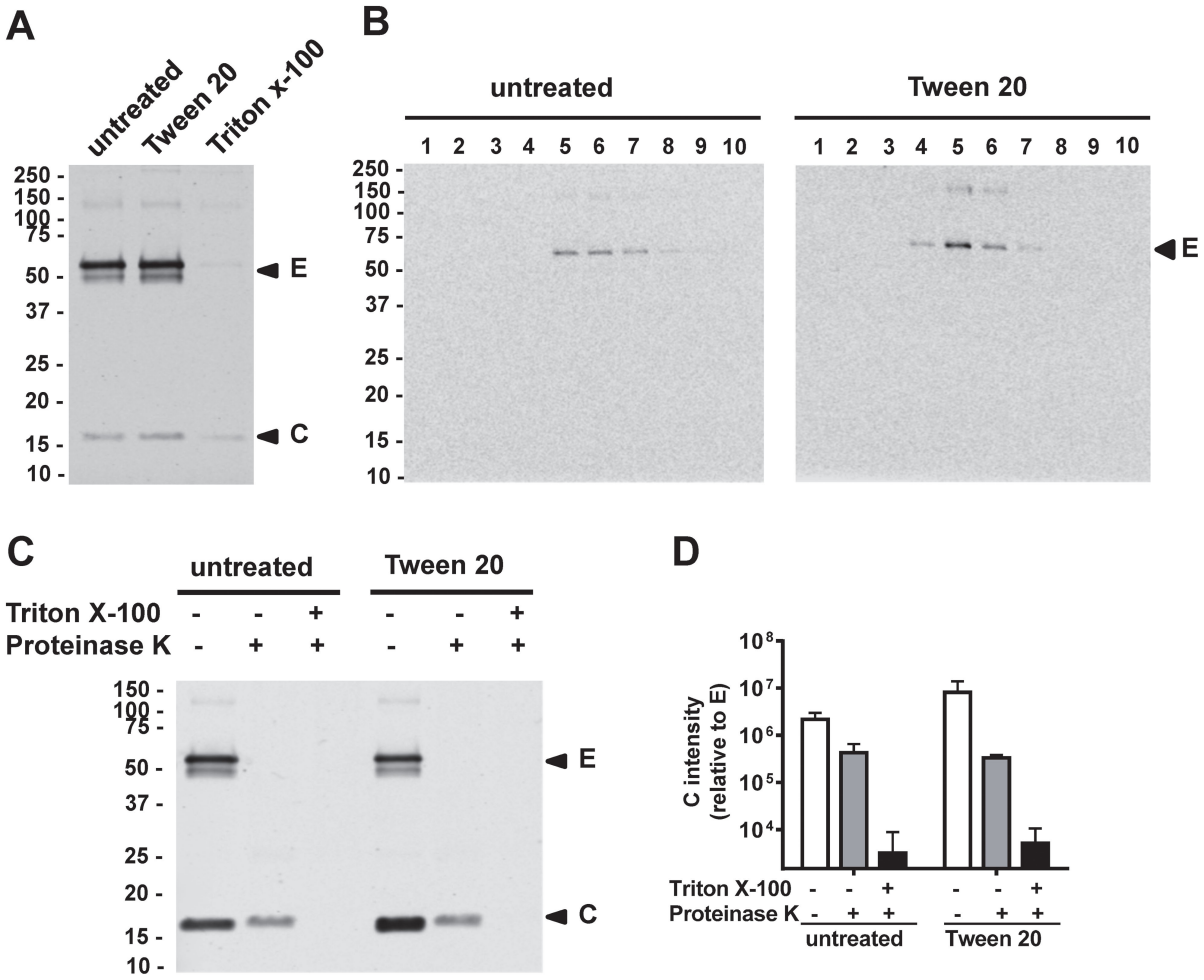
Membrane integrity of Tween 20-inactivated mDENV particles. **(A)** Culture supernatants of Vero-furin cells containing mDENV2 particles were untreated, treated with 1% Tween 20, or 1% Triton X-100 at 37 °C for 1 h, followed by 20% sucrose cushion ultracentrifugation. The pellets were subjected to Western blot analysis using anti-E (4G2) and anti-C (DB-32-40-30) mAbs. **(B)** Pellets containing Tween 20-inactivated or untreated mDENV1 particles were subjected to 15-60% sucrose gradient ultracentrifugation analysis; Each fraction was examined by Western blot analysis using DENV-immune human serum. **(C, D)** Proteinase K digestion-protection assay. Culture supernatants containing mDENV2 particles were untreated or treated with 1% Tween 20, followed by 20% sucrose cushion ultracentrifugation. The pellets were untreated or treated with 2% Triton X-100 before proteinase K digestion and subjected to Western blot analysis as in panel A **(C)**; the ratio of the intensity of C protein band to that of E protein band was quantified by Li-Cor Odyssey classic imager (LiCor Biosciences) with Image Studio software **(D)**. Data were means and standard deviations of 3 experiments. Representative Western blot analysis from 3 experiments was shown.

As traditional detergents emulsify lipid in the biological membranes, the membrane integrity of virus particles is usually disrupted after detergent treatment. To assess if Tween 20 inactivation affects viral membrane integrity, we examined mDENV2 particles. After treatment of virions with 1% Tween 20, the E and C proteins in partially purified virions were detectable by Western blot analysis at levels comparable to untreated controls (**Figure 4A**). In contrast, after treatment with a 1% solution of the commonly used non-ionic detergent Triton™ X-100, the E and C proteins were barely detectable. These findings suggest that Tween 20 inactivation, unlike Triton X-100 inactivation, does not fully disrupt viral membrane integrity. To further examine Tween 20-inactivated mature particles, we performed 15-60% sucrose gradient ultracentrifugation analysis (71). The peak of E protein from the Tween 20-inactivated mDENV1 particles was found in fractions 5 and 6 comparable to those of untreated particles (**Figure 4B**), suggesting Tween 20-inactivated mDENV1 particles maintained a similar density as untreated particles. In addition, proteinase K digestion-protection assay of Tween 20-inactivated mDENV2 particles revealed a pattern of complete digestion of E protein and protection of C protein, similar to that of untreated particles, further supporting the preservation of viral membrane integrity (70) (**Figures 4C, D**). As a control, prior treatment with 2% Triton X-100 led to complete digestion of E and C proteins (**Figure 4C**). Together these findings suggest that the membrane integrity of mDENV particles was not affected by 1% Tween 20 treatment.

### 3.5 Mechanisms of Tween 20 inactivation

**Figure 5:**
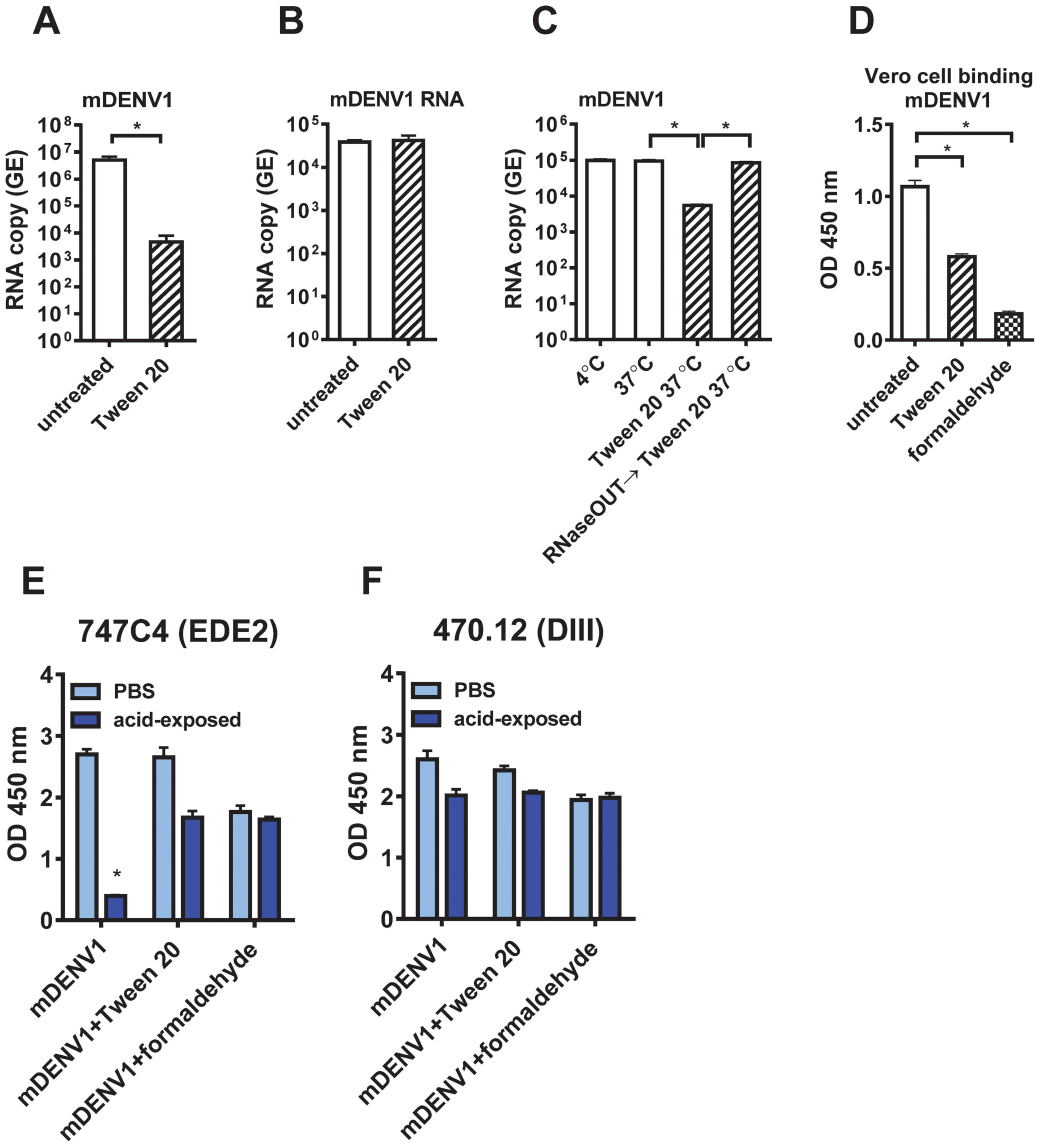
Mechanisms of Tween 20 inactivation. **(A)** Viral RNA extracted from 1 µg E-protein-equivalent of untreated and Tween 20-inactivated purified mDENV1 particles was subjected to real-time qRT-PCR to determine RNA GE copy numbers. **(B)** Viral RNA extracted from mDENV1 particles was treated with or without 1% Tween 20 at 37 °C for 1 h and subjected to qRT-PCR. **(C)** Culture supernatants containing mDENV1 particles were kept for 1 h at 4°C, 37°C, 37°C and treated with 1% Tween 20, or 37°C and treated with RNaseOUT and then 1% Tween 20, and subjected to viral RNA extraction, followed by qRT-PCR. **(D)** Vero cell-based virion binding ELISA. One µg E-protein-equivalent of purified untreated, Tween20- or formaldehyde-inactivated mDENV1 particles were added to Vero cells on ice for 2 h After washing and fixation with 4% paraformaldehyde, the bound particles were detected by ELISA using a DIII mAb (470.12). **(E, F)** Conformational changes of E protein on mDENV particles after low pH exposure. Purified untreated, Tween 20- or formaldehyde-inactivated mDENV1 particles were treated with MES buffer (pH 5.5) or PBS at 37°C for 1 h and then neutralized back to pH 7.2 by Tris buffer (pH 8.8). The PBS- or acid-exposed particles were examined by virion capture ELISA using an EDE2 (747C4) and DIII (470.12) human mAbs. Data were means and standard deviations of quadruplicates from one experiment. The two-tailed Mann-Whitney test was used to compare 2 groups. *P<0.05.

To investigate the mechanisms by which Tween 20 inactivates virus infectivity, we first used real-time quantitative reverse transcription polymerase chain reaction (qRT-PCR) to measure viral RNA in untreated or Tween 20-inactivated mDENV1 particles (72); Tween 20 treatment greatly (~3 log_10_ difference) reduced the amount of viral RNA (*P*<0.05) (**Figure 5A**). To examine whether Tween 20 can degrade RNA directly, purified viral RNA was treated with 1% Tween 20 at 37 °C for 1 h and quantified by qRT-PCR. Similar amounts of RNA were detected after Tween 20 treatment compared with the untreated control, confirming that Tween 20 does not directly degrade viral RNA (**Figure 5B**). To investigate if extracellular RNase is involved in viral RNA degradation, culture supernatants from DENV1-infected Vero cells were pre-treated with a potent noncompetitive inhibitor of pancreatic-type ribonucleases such as RNase A (RNaseOUT™ Recombinant Ribonuclease Inhibitor; Thermo Fisher Scientific) before Tween 20 inactivation. Compared with untreated control, Tween 20 inactivation resulted in 94.3% reduction (~2 log_10_ difference) of viral RNA, whereas pre-treatment with RNaseOut prevented such RNA degradation, suggesting extracellular RNase is involved in viral RNA degradation of Tween 20-inactivated DENV particles (**Figure 5C**). We further examined the infectivity of Tween 20-inactivated mDENV1 that was pre-treated with RNaseOut to preserve viral RNA and found no foci detectable (**Supplementary Figure 4**), suggesting Tween 20-dependent inactivation does not require degradation of the viral genome.

To examine if Tween 20-inactivated mDENV1 particles have reduced binding to target cells, we performed a Vero cells-based virion binding ELISA, and found a significant reduction in binding compared with untreated control particles (**Figure 5D**). As a comparison, a significant reduction in binding to Vero cells was also found in formaldehyde-inactivated mDENV1. During DENV entry, the acidic environment in the endosome triggers conformational changes of E protein during the transition from dimer to trimer which is required for membrane fusion. To investigate if Tween 20 affects such conformational changes of E protein, purified untreated, Tween 20- or formaldehyde-inactivated mDENV1 particles were exposed to pH 5.5 followed by neutralization back to pH 7.2, and examined by virion capture ELISA using a DIII mAb (470.12) and an EDE2 mAb (747C4) which has been shown to be sensitive to binding of acid-exposed DENV particles (21). Compared to PBS-exposed controls, untreated mDENV1 had a significant reduction in binding to 747C4 (14.7% remained) after acid exposure, whereas Tween 20- and formaldehyde-inactivated mDENV1 maintained 63% and 93.3% of binding to 747C4 (**Figure 5E**). As a control for mAb binding, untreated, Tween 20- or formaldehyde-inactivated mDENV1 had no significant reduction in binding to 470.12 after acid exposure (**Figure 5F**). These findings suggest that Tween 20 inactivation, like formaldehyde inactivation, prevents the conformational changes of E protein induced by acid exposure.

### 3.6 Tween 20-inactivated mDENV1 particles induce potently neutralizing antibodies

**Figure 6:**
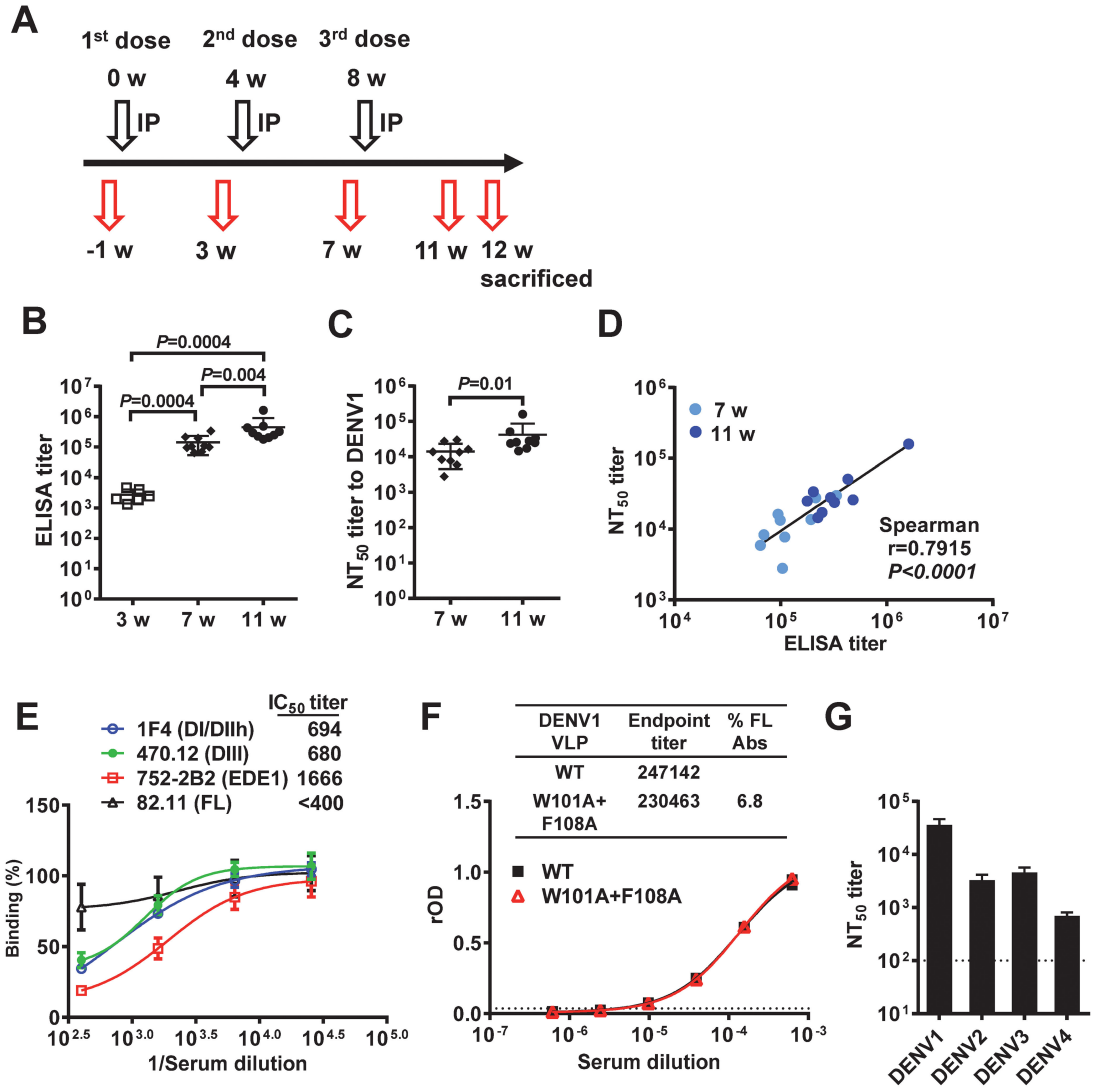
Immunogenicity of Tween 20-inactivated mDENV1 particles in BALB/c mice. **(A)** Immunization and blood sampling protocol: 6-10-week-old BALB/c mice (n=9, 5 females and 4 males) received 3 doses of 1 µg E-protein-equivalent of purified Tween 20-inactivated mDENV1 particles with 0.1% Alhydrogel (Alum) by intraperitoneal (IP) route at 0, 4 and 8 weeks; blood were drawn at 1 week pre-immunization and 3, 7 and 11 weeks post-immunization, and mice were sacrificed at 12 weeks. **(B)** Serum antibody endpoint titers were determined by ELISA coated with purified mDENV1 virions. **(C)** Serum NT_50_ titers to DENV1 at 7 and 11 weeks were determined by microneutralization test. **(D)** The relationship between NT_50_ titers and ELISA endpoint titers (two tailed Spearman correlation test). **(E)** Serial dilutions of pooled immune sera at 11 weeks were tested for blockade of binding ELISA using human mAbs including 1F4 (DI/DII-hinge), 470.12 (DIII), 752-2B2 (EDE1) and 82.11 (FL). **(F)** Pooled immune sera at 11 weeks were examined for binding to DENV1 WT and FL mutant (W101A+F108A) VLPs by ELISA. Endpoint titers and % FL antibody were determined (74). **(G)** NT_50_ titers to DENV1–4 of pooled immune sera at 11 weeks. Data were means and standard deviations of quadruplicates from one experiment. The two-tailed Mann-Whitney test was used to compare 2 groups.

To evaluate the immunogenicity of Tween 20-inactivated mDENV1 particles, 6-10-week-old BALB/c mice (n=9) were immunized with 3 doses of 1 µg E-protein-equivalent of purified Tween 20-inactivated mDENV1 particles with a four-week interval between doses (**Figure 6A**). Binding and neutralizing antibody titers to DENV1 increased significantly after each dose, and a positive correlation was found between binding and neutralizing antibodies (**Figures 6B–D**). To examine the quality of antibodies induced, pooled immune sera at 11 weeks were analyzed by a blockade of binding ELISA using different human mAbs (**Figure 6E**). While a dose-dependent inhibition of binding was observed for all four mAbs tested, a significant inhibition (>50%) was only observed for three potently neutralizing mAbs (DI/DII-hinge, DIII and EDE1) but not the weakly neutralizing FL mAb, with high 50% inhibition concentration (IC_50_) titers to potently neutralizing mAbs, in particular the EDE1 mAb 752.2B2 (IC_50_ titer: 1666). We further examined the binding of pooled immune sera to DENV1 WT or FL-mutant VLPs and determined the proportion of FL antibody, which was 6.8% and much lower than that reported in human sera after DENV infection (74) (**Figure 6F**). Taken together, these findings suggest that Tween 20-inactivated mDENV1 predominantly induced antibodies that can block potently neutralizing human mAbs and minimally induced FL antibodies, which are weakly neutralizing and can potentially cause ADE. Notably, pooled immune sera at 11 weeks neutralized viruses of all four DENV serotypes (NT_50_ titers to DENV1, DENV2, DENV3 or DENV4: 35353, 3191, 4449, or 678, respectively) (**Figure 6G**), which is consistent with the presence of EDE1-like antibodies with the highest IC_50_ titer and suggests that Tween 20-inactivated mDENV1 particles could induce broadly neutralizing antibody responses against four DENV serotypes.

## 4 Discussion

In this study, we explored the feasibility of employing Tween 20 inactivation of mDENV particles to develop a new vaccine candidate antigen. Based on binding assays with different panels of well-characterized human anti-DENV mAbs, we found that Tween 20 inactivation preserved the epitopes recognized by potently neutralizing mAbs better, as compared to formaldehyde and UV inactivation. Moreover, mice immunized with Tween 20-inactivated mDENV1 particles generated antibody responses that could block most potently neutralizing mAbs and neutralize all four DENV serotypes. At the same time, there was minimal FL recognition. To our knowledge, this is the first report showing Tween 20-inactivated mDENV particles represented a potential DENV vaccine immunogen.

It is worth noting that the relative binding of mDENV1 to all 14 mAbs tested was higher after Tween 20 inactivation (50-100%) compared with binding after formaldehyde (37-85%) or UV (16-90%) inactivation (*P* = 0.04 or *P* = 0.0001, respectively, the two-tailed Wilcoxon signed rank test, **Figure 2F**). This finding was mainly due to higher relative binding to quaternary epitope mAbs after Tween 20 inactivation than formaldehyde or UV inactivation (*P* = 0.03, both comparisons, the two-tailed Wilcoxon signed rank test, **Figure 2F**). In contrast, there was no difference in the relative binding to DIII mAbs between Tween 20 and formaldehyde or UV inactivation (*P* = 0.25 or *P* = 0.50, respectively, the two-tailed Wilcoxon signed rank test, **Figure 2F**). DIII neutralizing mAbs are mainly TS and have been shown to contribute to protection against a single flavivirus, such as WNV, JEV or TBEV (78–80); these may explain the protective effects of formalin-inactivated TBEV, JEV and WNV vaccines in humans and animals.

The mode of action studies suggested that multiple mechanisms are involved in Tween 20 inactivation. Based on sucrose-gradient ultracentrifugation and protein K digestion-protection experiments, we showed that Tween 20 does not significantly disrupt viral membrane integrity, which may contribute to the preservation of epitopes, especially quaternary epitopes. Moreover, Tween 20 treatment led to reduced virus binding to target cells and prevented acid-induced conformational changes in the E protein, suggesting both attachment and post-attachment steps of virus entry are involved. Unexpectedly, Tween 20 treatment of virions resulted in a 2 to 3 log_10_ reduction of RNA copy number, which did not occur when virions were pre-treated with an RNAse inhibitor (RNaseOut), suggesting the involvement of extracellular RNase. This finding not only explains the effectiveness of Tween 20 inactivation on virions but also provides new insight into its mode of action. We hypothesize that Tween 20 penetrates the lipid bilayer membrane, allowing extracellular RNase to degrade viral RNA inside the particles, while maintaining substantial membrane integrity with rigidity of the lipid bilayer, thus preventing conformational changes of E protein in acidic environments. Future studies involving high-resolution cryo-EM reconstruction in combination with other methods, or conventional transmission electron microscopy (TEM) imaging on Tween 20-inactivated particles may help to delineate these possibilities and could provide important insights in the physical characterization of the particles.

The immunogenicity study in mice revealed Tween 20-inactivated mDENV1 particles induced high titers of binding and neutralizing antibodies. Assessing the quality of antibodies revealed that they contain minimal FL antibodies and primarily antibodies that can block three potently neutralizing mAbs (DI/DII-hinge, DIII and EDE1) with the highest IC_50_ titer (1666) against 752.2B2, a EDE1 mAb. Consistent with this finding, the induced antibodies can broadly neutralize representative viruses from all four DENV serotypes. Taken together, these findings support the notion that mDENV particles represent a new vaccine candidate immunogen to induce potently neutralizing antibodies with reduced risk of ADE. Following this proof-of-concept study, challenge experiments are needed to show the protective effect of Tween 20-inactivated mDENV particles *in vivo*.

It should be noted that the neutralizing antibodies induced by Tween 20-inactivated mDENV1 particles were not equally balanced against all four DENV serotypes (NT_50_ titers: highest to DENV1 and lowest to DENV4) (**Figure 6G**). As the neutralizing antibodies wane over time especially those against DENV4, a potential ADE may occur upon infection with non-DENV1 viruses, such as DENV4. Therefore, tetravalent Tween 20-inactivated mDENV particles rather than monovalent Tween 20-inactivated mDENV1 particles are likely to be the final vaccine candidate. Previous studies have shown the maturation status of DENV varies with cell types (24, 36); for example, DENV derived from dendritic cells, one of the target cells of DENV *in vivo*, contained more mature particles, whereas DENV grown in C6/36 cells, a mosquito cell line, were predominantly (more than 50%) immature particles. Studies of mechanisms of flavivirus neutralization revealed that the potency of neutralizing mAbs is affected by the maturation status of particles (29, 30). It is possible that the potently neutralizing antibodies induced by mDENV particle vaccine neutralize mature particles efficiently, but neutralize immature particles, such as DENV coming from mosquito vector, less efficiently. However, a recent study showed that potently neutralizing mAbs, which recognize quaternary epitopes, can neutralize both mature particles and C3/36-derived DENV particles (predominantly immature particles) well, compared with FL or bc loop mAbs (28). Moreover, after a few rounds of replication *in vivo*, including dendritic cells and mononuclear cells, newly produced DENV particles become predominantly mature particles (28), which will be susceptible to efficient neutralization by potently neutralizing antibodies induced by mDENV particle vaccine.

There are several limitations of this study. First, due to the availability of well-characterized human mAbs, our epitope study only included a limited number of mAbs and focused mainly on Tween 20-inactivated mDENV1 and mDENV2 particles. Future studies including a larger number of human mAbs and those recognizing other DENV serotypes are needed to better understand the epitope preservation of mDENV3 and mDENV4 particles after Tween 20 inactivation. Second, our study only compared the epitope preservation between Tween 20 and formaldehyde or UV inactivation. Future studies including other inactivation methods such as H_2_O_2_ and psoralen inactivations would provide a comprehensive understanding of epitope preservation by different inactivation methods. Third, as Tween 20-inactivated mDENV1 particles, which keep the same maturation status to induce the favorable immune profile, are promising for future vaccine study in non-human primates or humans, we did not test non-inactivated mDENV1 control as a protein-based immunogen in our immunocompetent mice to address whether Tween 20 inactivation is necessary to elicit the favorable immune profile.

In conclusion, this proof-of-principle study shows that Tween 20-inactivated mDENV1 particles better preserved the epitopes recognized by potently neutralizing mAbs as compared with other methods. The antibodies induced in mice by immunization with these particles not only showed high titers of binding and neutralizing activities but also the desirable qualities of recognizing the FL antigenic site only minimally, competing for binding with several potently neutralizing and protective mAbs, and neutralizing viruses of all four DENV serotypes. The studies support the conclusion that inactivation of mature DENV particles with Tween 20 is a promising strategy for generating new immunogens that could be incorporated into new inactivated DENV vaccine candidate formulations.

## Data Availability

The original contributions presented in the study are included in the article/**Supplementary Material**. Further inquiries can be directed to the corresponding author.
