# Supplementary material for: Mature dengue virus particles inactivated by a non-ionic detergent retain quaternary neutralizing epitopes and induce potent neutralizing antibodies
Source: Front Immunol. 2025 Sep 10;16:1626823. doi: 10.3389/fimmu.2025.1626823 (PMC12457450; doi:10.3389/fimmu.2025.1626823)
Supplement: Supplementary file 1 [file DataSheet1.pdf]

FS1

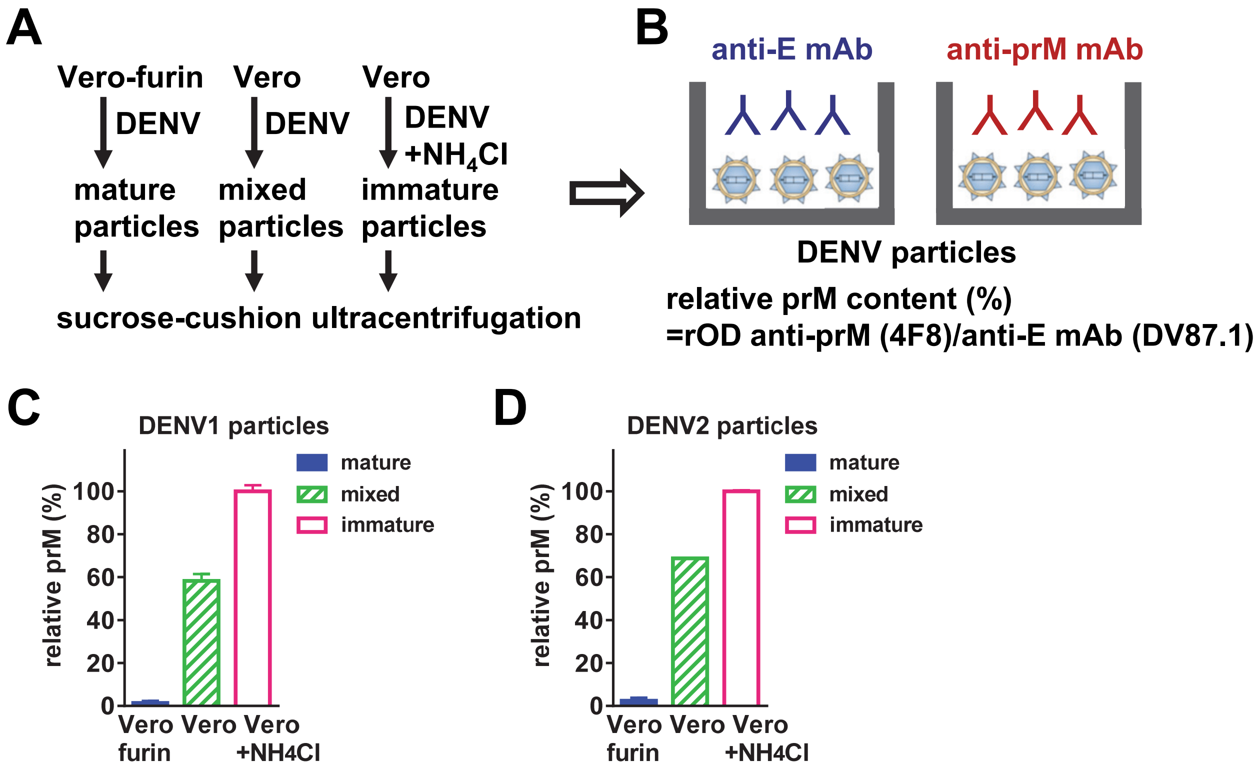

Supplementary Figure 1

Relative prM content of mature DENV particles in comparison with mixed and immature DENV particles. (A) Generation of mDENV, mixed DENV and immature DENV from Vero-furin cells, Vero cells, and Vero cells in the presence of ammonium chloride, respectively (31,63), followed by sucrose-cushion ultracentrifugation. (B) Different DENV particles were coated on ELISA plates, followed by detection with human anti-prM (4F8) and anti-E (DV87.1) mAbs. The relative prM content (%) equals the ratio of rOD of anti-prM mAb to that of anti-E mAb as described previously (31). (C,D) The relative prM content (%) of different DENV1 (C) and DENV2 (D) particles.

FS2

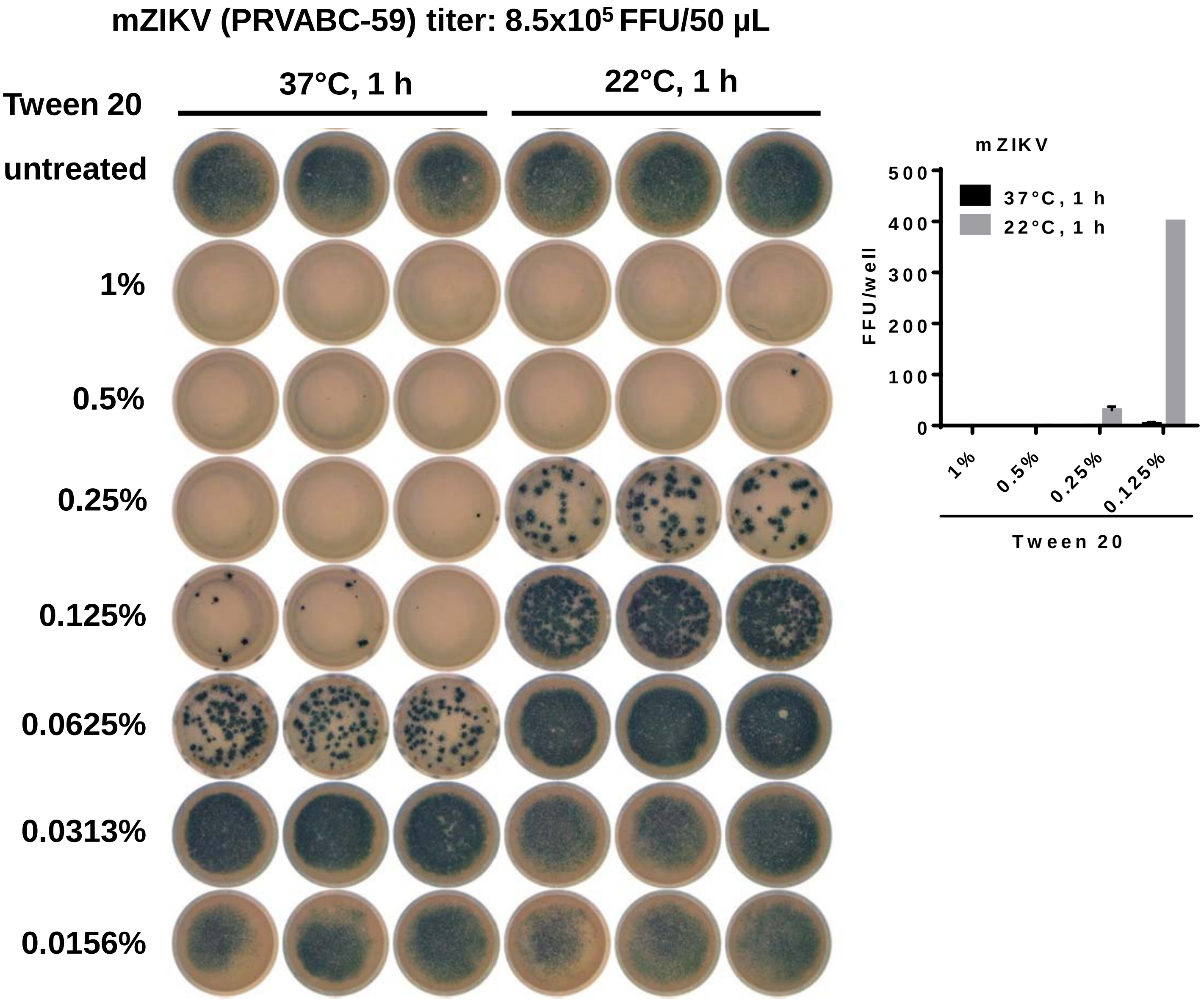

Supplementary Figure 2

Inactivation of mZIKV particles with different concentrations of Tween 20 at 22°C and 37°C. mZIKV (8.5×10^5 FFU, PRVABC-59 strain) was inactivated by Tween 20 at 22°C or 37°C for 1 h. Inactivated mZIKV particles were 10-fold serially diluted and inoculated into Vero cells in triplicates for 48 h, followed by fixation and staining with mouse anti-E mouse mAb (4G2), secondary antibody and TrueBlue as described in the Methods. FFU per well were shown at the right.

FS3

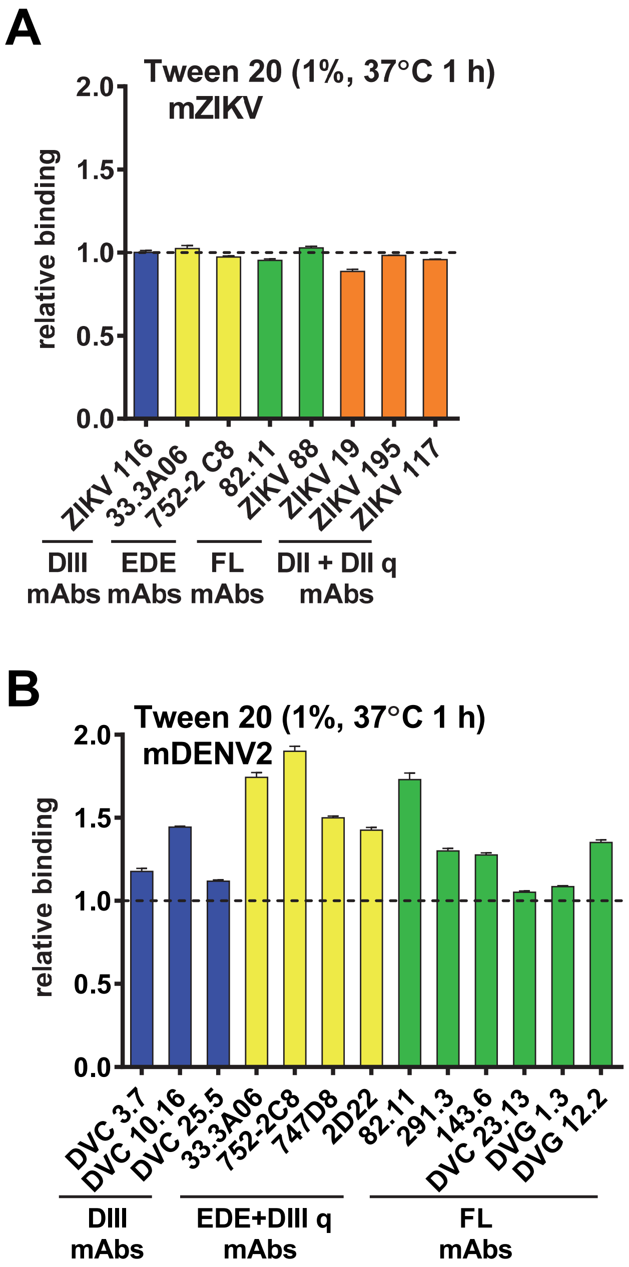

Supplementary Figure 3

Epitope preservation of inactivated mZIKV and mDENV2 particles. Virion-capture ELISA was used to assess the epitopes on Tween 20-inactivated mZIKV particles (A) using 8 human mAbs including four groups: DIII (blue), EDE (yellow), FL (green), DII and DII quaternary (DII q) (orange) epitope mAbs, and epitopes on Tween 20-inactivated mDENV2 particles (B) using a panel of 13 human mAbs including three groups: DIII (blue), EDE and DIII quaternary epitope (DIII q) (yellow), and FL (green) mAbs (Table 1). The relative binding was the OD of a mAb bound to inactivated mZIKV or mDENV2 particles relative to that to untreated particles. Data were means and standard deviations of triplicates from one representative experiment of two.

FS4

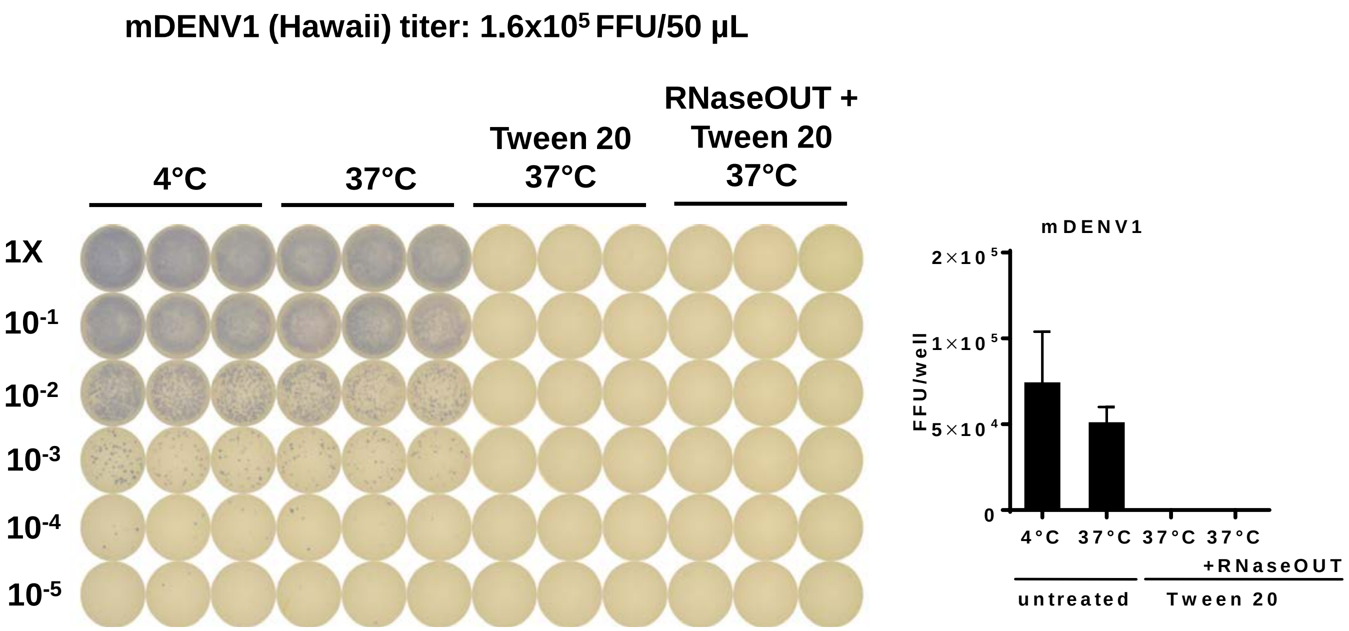

Supplementary Figure 4

Lack of infectivity of Tween 20-inactivated mDENV1 particles that was pre-treated with RNaseOut. mDENV1 (1.6x10^5 FFU, Hawaii strain) were untreated (at 4°C or 37°C for 1 h), treated with 1% Tween 20 at 37°C for 1 h, or pre-treated with RNaseOut, followed by 1% Tween 20 at 37°C for 1 h, and then 10-fold serially diluted and inoculated into Vero cells in triplicates for 48 h, followed by fixation and staining with mouse anti-E mouse mAb (4G2), secondary antibody and TrueBlue as described in the Methods. FFU per well were shown at the right.
